# Machine Learning and Artificial Intelligence in PK‐PD Modeling: Fad, Friend, or Foe?

**DOI:** 10.1002/cpt.3165

**Published:** 2024-01-05

**Authors:** Zhonghui Huang, Paolo Denti, Hitesh Mistry, Frank Kloprogge

**Affiliations:** ^1^ Great Ormond Street Institute of Child Health University College London London UK; ^2^ Division of Clinical Pharmacology, Department of Medicine University of Cape Cape Town South Africa; ^3^ Division of Pharmacy University of Manchester Manchester UK; ^4^ Institute for Global Health University College London London UK

Developing pharmacokinetic‐pharmacodynamic (PK‐PD) models requires a significant amount of time from highly skilled scientists and the demand for this expertise far outstrips the current supply. The use of machine learning (ML) and artificial intelligence (AL) in PK‐PD modeling promises to reduce the number human supervision hours and improve predictive performance, but in its current form it suffers from various limitations. In this perspective, we aimed to structure the main trends and define boundaries and opportunities.

## MACHINE LEARNING AND PK AND PK‐PD MODEL DEVELOPMENT

PK‐PD modelers leverage various AI‐based tools in their daily routine and this has without doubt contributed to increased productivity. For example, AI‐driven programming tools can help generating code, for example, Git‐Hub Co‐Pilot, presenting results, for example, Microsoft Co‐Pilot, and scientific writing, such as Large Language models. However, unlike in other fields, AI/ML has not yet been integrated into a PK‐PD modeler's daily routine for analysis of time series data.

Currently, most efforts present proof of concepts, focused on adopting methodologies used in other fields. Broadly speaking, current applications of ML to PK‐PD modeling can be divided into two main streams. The first aims to predict individual observations, exposures, etc., using purely data‐driven AI algorithms, that is, using AI as a substitute for the conventional model‐based approach encompassing a structural model, variability, and covariate effects. The second does not substitute the conventional model‐based approach, but it rather aims at using AI algorithms to guide, inform, and expedite the development of nonlinear mixed‐effect model structures and parameter‐covariate relations. Thus, the focus is on increasing efficiency and not moving away from the familiar models we currently use (e.g., ordinary differential equations or analytical solutions thereof), which are grounded in our knowledge of biology, physiology, and pharmacology.

Here, we aimed to structure the main trends in published AI/ML research, applied to PK‐PD, and define present boundaries and opportunities.

## ML AS SUBSTITUTE OF CONVENTIONAL STRUCTURAL AND STOCHASTIC MODELS

Algorithms that are members of the neural network and tree‐based families are reported to display good fits for individual observations in PK and PD time series data. Likewise, it is commonly known that spline models fit PK and PD time series data well.

What both aforementioned ML and spline models have in common is that they are driven solely by the experimental data they are fit on. There is a complete absence of a mechanistic or semimechanistic structure relatable to conceptual biological knowledge, such as the primary PK concepts of absorption, distribution, and elimination. There is, however, broad consensus that having these conceptual biological priors embedded in our PK‐PD models enables meaningful characterization of the variability between patients. Kreutzner *et al*. 2022, for instance, showed that AI methods performed inferior to conventional population PK (PopPK) models in quantifying random variability for their example.^1^

Furthermore, because no plausibility and consistence with physiology, biology, or pharmacology is embedded in these types of ML models, out of sample predictions are unreliable. For example, prediction of concentrations at times beyond those observed in the clinical study or simulation of exposure at dosing regimens that were not studied appear to be a challenge.^2^, ^3^ Until this issue is addressed, ML algorithms describing PK and PD time series data cannot be used for dose/schedule optimization or extrapolation into unseen or poorly studied populations.

Given that this is one of the core reasons for quantitative pharmacologists to design, generate, and analyze PK and PD time series data, efforts in using ML techniques to entirely substitute our conventional PK and PD models seem misguided. However, hybrid ML/PK approaches have been reported with the aim to maintain the predictive potential of AI/ML while compensating their lack of biological prior by integrating a traditional PopPK model.^4^, ^5^, ^6^

## ML ASSISTED MODELING: STRUCTURAL AND STOCHASTIC MODEL SELECTION

ML methods have also been used to select structural PK, PD, and covariate models. Typically, metaheuristic algorithms are used to guide these processes. This family of algorithms can be divided into single‐solution vs. population‐based searches, although to the best of our knowledge single‐solution algorithms, like simulated annealing, have not been applied to PK‐PD model building. On the other hand, population‐based metaheuristic models including evolutionary algorithms, for example, genetic algorithm, and swarm intelligence algorithms, such as ant colony optimization, have been reported to perform well. Deployment of Neural‐ordinary differential equations (ODEs) has also been reported with good ability to fit PK data.^7^ However, this method might only be meaningful for late‐stage clinical or postmarketing research on population variability and precision dosing as the amount of data that is needed to support these algorithms is never available in early phases of development.

ML assisted model building techniques use fitness functions, a purely objective approach, to prioritize competing models but the design of an optimal fitness function has not yet been explored in depth. This has been reported to translate into bias toward selection of simple models,^8^ and poorly selected initial estimates might lead to poor accuracy on clearance and volume of distribution estimates.^9^

Furthermore, the number of models to be tested with AI methods is much larger compared with conventional PK and PD model development strategies, due to the failure to implement a substitute of the human visual inspection component into ML assisted model building algorithms. Unsurprisingly, the error rates are also inflated compared with conventional stepwise model building. This results in an inefficient way to recover the model structure best fitting the data, which does not comply with Green Coding principles, and there is a broad consensus that human supervision remains needed.

However, future improvements in the implementation of the algorithms mentioned above should be able to address some of their problems and it is plausible that these techniques will gradually start contributing to improving the productivity of pharmacometric modelers even further.

## ML‐ASSISTED MODELING: COVARIATE AND PHARMACODYNAMIC MODEL DEVELOPMENT

ML‐assisted identification of covariate‐parameter relations and PD readout/biomarker has also been reported. These may include unsupervised and supervised ML methods for exploration of unknown covariate effects and hypothesis testing of covariate‐parameter relationships.

These methods may be useful to offer insight on temporal trends in biomarkers, multifactorial relationships, for example, nonlinear relationship visible only in subgroups, or other phenomena for which we do not have a mechanistic description yet. However, results should be considered a starting point for further exploration, hypothesis testing, and implementation of mechanism‐based models, much like when a modeler observes a trend in a plot and needs to decide how to proceed. Various ML applications across medical specialties concluded that poor methodological quality comes with an increased risk of bias.^10^ Especially for PK and PK‐PD, caution is required given that PK and PK‐PD datasets are typically small, resulting in potential failure to prevent overfitting.

## LIMITATIONS AND OPPORTUNITIES

As a new and powerful tool, we encourage the use of AI/ML in the field of PK‐PD modeling. AI and ML represent a great addition to the toolbox available to PK‐PD modelers. They can be used to quickly identify trends and associations within a dataset, even very large. This can save a significant amount of time and allow for the identification of patterns in the data that would escape the most experienced eye.

However, like all other modeling approaches that are entirely data‐driven, AI and ML generate models that, while fitting the data well, bear in general no relationship with the phenomenon they aim to describe. This means that they cannot be relied upon to distinguish association and causation or in terms of their predictive capabilities, especially outside of the range used for their creation. Prediction of unobserved scenarios is one of the main reasons why we develop conventional population PK and PD models, so this flaw is a major limitation.

In summary, entirely unsupervised use of AI and ML, especially as a substitute for structural models, is therefore not encouraged and unlikely to offer a credible alternative to mechanism‐based models. However, AI‐ and ML‐aided model development is an attractive and powerful tool that may expedite the work of modelers.
